# Synthesis and Molecular Modeling Studies of *N′*-Hydroxyindazolecarboximidamides as Novel Indoleamine 2,3-Dioxygenase 1 (IDO1) Inhibitors

**DOI:** 10.3390/molecules22111936

**Published:** 2017-11-09

**Authors:** Dong-Ho Lee, Joo-Youn Lee, Jieun Jeong, Miok Kim, Kyung Won Lee, Eunseo Jang, Sunjoo Ahn, Chang Hoon Lee, Jong Yeon Hwang

**Affiliations:** 1 Bio & Drug Discovery Division, Korea Research Institute of Chemical Technology, Daejeon 34114, Korea; ldh1125@krict.re.kr (D.-H.L); chemworld@hanmail.net (J.J.); miok@krict.re.kr (M.K.); ek00322@krict.re.kr (K.W.L.); eunseooo@krict.re.kr (E.J.); sahn@krict.re.kr (S.A.); 2 Department of Chemistry, Sogang University, Seoul 121-742, Korea; 3 Korea Chemical Bank, Korea Research Institute of Chemical Technology, 141 Gajeong-ro, Yuseong-gu, Daejeon 34114, Korea; leejy@krict.re.kr; 4 Immunotherapy Convergence Research Center, Korea Research Institute of Bioscience and Biotechnology (KRIBB), Daejeon 34141, Korea; 5 Department of Medicinal Chemistry and Pharmacology, University of Science & Technology, Daejeon 34113, Korea

**Keywords:** indoleamine 2,3-dioxygenase 1, *N*′-hydroxyindazolecarboximidamide, cancer immunotherapy, tryptophan depletion, kynurenine production

## Abstract

Indoleamine 2,3-dioxygenase 1 (IDO1) is an immunosuppressive enzyme that is highly overexpressed in various cancer cells and antigen-presenting cells. It has emerged as an attractive therapeutic target for cancer immunotherapy, which has prompted high interest in the development of small-molecule inhibitors. To discover novel IDO1 inhibitors, we designed and synthesized a series of *N′*-hydroxyindazolecarboximidamides. Among the compounds synthesized, compound **8a** inhibited both tryptophan depletion and kynurenine production through the IDO1 enzyme. Molecular docking studies revealed that **8a** binds to IDO1 with the same binding mode as the analog, epacadostat (INCB24360). Here, we report the synthesis and biological evaluation of these hydroxyindazolecarboximidamides and present the molecular docking study of **8a** with IDO1.

## 1. Introduction

Indoleamine 2,3-dioxygenase 1 (IDO1) is a monomeric heme-containing enzyme that catalyzes the oxidation of l-tryptophan to *N*-formylkynurenine [1,2]. *N*-Formylkynurenine is then converted via the kynurenine pathway to a series of biologically-active metabolites including l-kynurenine, kynurenic acid, 3-hydroxykynurenine, xanthurenic acid, anthranilic acid, and quinolinic acid. These metabolites inhibit the proliferation and differentiation of T cells, leading to immune tolerance [3,4,5]. Overexpression of IDO1 has been observed in various cancer cells and antigen-presenting cells, and depletion of IDO1 has been shown to inhibit tumor growth and metastases [6,7,8,9]. Therefore, IDO1 has emerged as an attractive therapeutic target for cancer immunotherapy [10].

**Figure 1:**
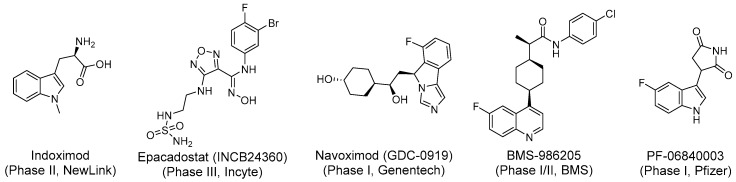
Structure of IDO1 inhibitors in clinical trials.

A number of IDO1 inhibitors have been reported in the literature and several of these compounds are being investigated in clinical trials for use in combination therapy with other therapeutic interventions (Figure 1) [11,12].

Among the IDO1 inhibitors currently being studied, two advanced compounds, INCB24360 (epacadostat) [13] and GDC-0919 (Navoximod) [14,15], were found to be highly potent in both enzymatic and cellular assays. The X-ray crystal structure of cmpd-24 (GDC-0919 analog) revealed that the imidazoleisoindole core is located in pocket A, while the nitrogen atom of imidazole coordinates with the heme iron [16]. The 1-cyclohexylethanol residue extends to pocket B and forms hydrophobic interactions with Phe226, Ile354, and Leu384. Interestingly, compd-24 forms two additional intramolecular hydrogen bonds with the IDO1 enzyme. The nitrogen atom in isoindole binds to the hydroxyl group found in the cyclohexylethanol residue, while the hydroxyl group of cmpd-24 forms an intramolecular hydrogen bond with the 7-propionic acid of the heme iron to increase IDO1 binding affinity. In the case of INCB24360, its crystal structure and predicted model study suggest that the hydroxyl group in the oxime moiety exists in the *cis*-conformation to bind to IDO1 and forms the observed intramolecular hydrogen bonding network [13,17]. Recently, the X-ray crystal structure of IDO1 with INCB14943 (epacadostat analog, PBD:5XE1) has also been reported. A nitrogen in the oxime moiety of INCB14943 covalently coordinates with the heme iron, and a phenyl ring, situated in pocket A, forms hydrophobic interactions with V130, F164, and L234. An intermolecular hydrogen bond also forms between the NH_2_ in the furazan moiety and the side chain of S263 in IDO1. In addition, the hydroxyl group in the oxime moiety is essential for intramolecular hydrogen bond networks between the NH_2_ group in the furazan moiety and the NH moiety of INCB14943 [13,18].

**Figure 2:**
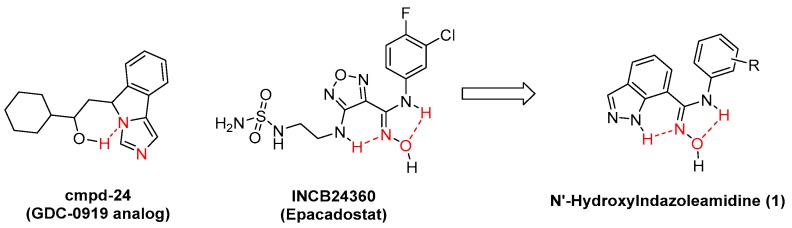
Rational design of novel IDO1 inhibitor (**1**) from the known molecules.

As part of our efforts to identify a novel IDO1 inhibitor for immune-oncology drug discovery, we designed novel *N′*-hydroxyindazolecarboximidamides **1**, in which the indazole moiety is a surrogate of furazan in epacadostat (Figure 2). Herein, we report the synthesis and anti-IDO1 activities of the designed compounds and discuss the molecular docking study of the selected compound.

## 2. Results and Discussion

### 2.1. Chemistry

**Scheme 1:**
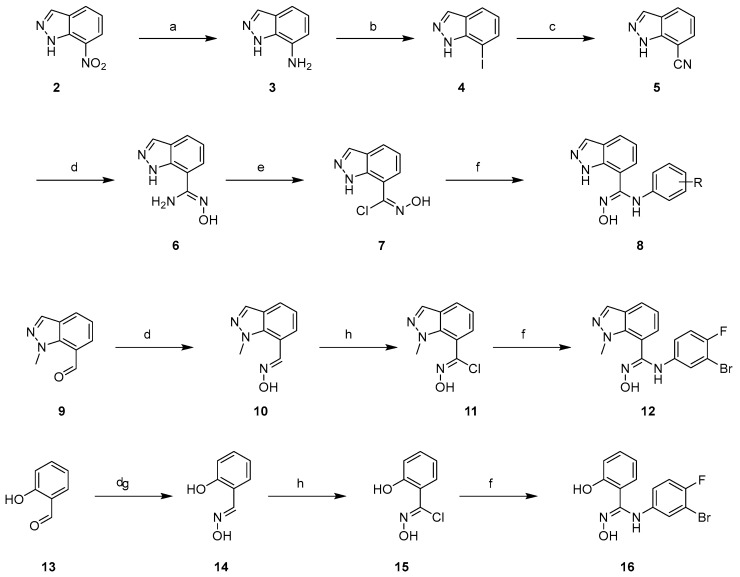
Synthesis of compounds **8**, **12** and **16**. *Reagents and conditions*: (**a**) 10% Pd/C, H_2_, EtOH, r.t.; (**b**) NaNO_2_, H_2_O, HCl, H_2_SO_4_, KI; (**c**) Zn(CN)_2_, Pd_2_(dba)_3_, dppf, Zn, DMA, reflux; (**d**) hydroxylamine hydrogen chloride, NaHCO_3_, H_2_O, EtOH, 85 ^°^C; (**e**) (i) 6N HCl, H_2_O, AcOH, 45 °C, (ii) HCl, NaNO_2_, H_2_O, 0 °C, 3 h; (**f**) amines, NaHCO_3_, H_2_O, THF, 60 °C; (**g**) NCS, DMF, r.t., 6 h.

*N′*-Hydroxyindazolecarboximidamides were synthesized as described in Scheme 1, starting from commercially-available 7-nitroindazole **2**. The nitro group in **2** was reduced to amine **3** using Pd/C in a hydrogen atmosphere. The amino group in **3** was transformed to iodine by treatment of NaNO_2_ and KI [19]. Introduction of nitrile in **5** was accomplished by treatment of Zn(CN)_2_, Pd_2_(dba)_3_, 1,1′-bis(diphenylphosphino)ferrocene (dppf), and Zn [19]. Treatment of hydroxylamine in **5** afforded hydroxylamidine **6**, which was converted to *N*-hydroxyimidoyl chloride **7** by Sandmeyer conditions with NaNO_2_ and HCl [13]. The *N*-hydroxyimidoyl chloride was substituted with the desired hydroxyamidines **8** by treatment of the corresponding amines. *N*-Methylindazole **12** was synthesized from the commercially-available *N*-methylindazole carboxaldehyde **9**, which was treated with hydroxylamine to afford oxime **10**. Oxime **10** was oxidized with *N*-chlorosuccinimide (NCS) to afford *N*-hydroxyimidoyl chloride **11 [20]**, which was converted to the desired **12** by treatment of the amine. 2-Hydroxyphenyl **16** was also prepared from 2-hydroxybenzaldehyde **13** by the same synthetic method described above (for the creation of **12**).

### 2.2. Biological Evaluation

All synthesized compounds were evaluated for inhibitory activities against IDO1. For this purpose, we generated human IDO1 gene-expressing HEK293 recombinant cells and used those cells to analyze the inhibitory capacity of the compounds. Briefly, we induced the expression vector system of IDO1 by treating HEK294 cells with doxycycline, and measured both the depletion of l-tryptophan in culture media and the generation of kynurenine in IDO1-overexpressing HEK293 cells by using the LC-MS system upon culture supernatants.

The synthesized compounds were tested at a concentration of 10 μM for IDO1-inhibitory activities in cell-based assays measuring levels of tryptophan and kynurenine (Table 1). Among the compounds, **8a**–**c** displayed >50% inhibition against IDO1, whereas others were almost inactive at 10 µM concentration. These selected active compounds were tested to determine IC_50_ values by dose-response curve (DRC) analysis (Table 1), and they exhibited moderate inhibitory activity ranges in both tryptophan depletion and kynurine production. These actives possess 3-bromo or 3-chloro substitution in the phenyl group, and the compounds with an additional 4-fluoro substitution showed increased anti-IDO1 activity. 3-Bromo-4-fluorophenyl **8a** was the most active in this series. Some active compounds (**8a**–**c**) in the cell-based assay were further tested for hIDO1 inhibition by enzymatic assay and displayed hIDO1 inhibitory activities. It was reported that the substituent of aniline in the A-pocket is very important for IDO1 inhibition [13]. For example, fluoro- or no substituted compound in the *para* position was active, while others were almost inactive. In addition, meta-halogen substituents in aniline, including chloro- and bromo, were required for the activity. Thus, our results almost match those of a previous report. The intra- and intermolecular hydrogen bonding network in the active site of IDO1 plays an important role in their inhibitory activity, as mentioned earlier. We presumed that the *N*-hydrogen in the indazole moiety could involve a hydrogen bonding complex in the active site of IDO1. To confirm this hypothesis, *N*-methylindazole compound **12** was evaluated. As expected, it did not inhibit IDO1 in both cell-based and enzymatic assays. Interestingly, phenol **16**, which consists of a bioisosteric replacement of indazole and bears a hydroxyl group for hydrogen bonding, showed moderate inhibitory activity. This result strongly supports the hypothesis that a hydrogen bonding network is critical for IDO1 inhibition.

### 2.3. Molecular Docking Study

**Figure 3:**
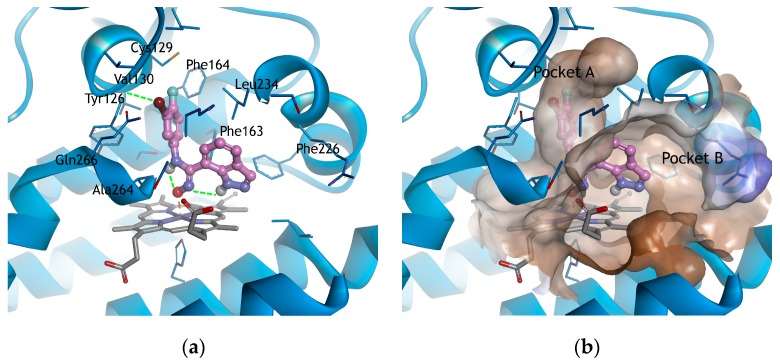
Docking model **8a** against IDO1. (**a**) Binding mode of **8a** (pink, ball and stick style) coordinated to heme; (**b**) The surface model of the active site bound to **8a** is colored because of hydrophobicity. Hydrogen bonds of inter- and intra-molecular interactions are shown as green dashed lines and interaction residues are represented by the stick model.

To understand the binding mode of **8a** for IDO1, docking studies were performed using the IDO1 X-ray crystal structure in complex with Amg-1 (PBD code 4PK5) [21]. The proposed binding model of **8a** was very similar to that of epacadostat (Figure 3a) [17]. The hydroxyl group of the hydroxyamidine moiety in **8a** forms a covalent bond with the heme iron, and the 3-Br and 4-F substituted phenyl groups bind into the hydrophobic pocket A, which consists of Tyr126, Cys129, Val130, Phe163, Phe164, Leu234, Gly262, and Ala264. This proposed binding conformation of **8a** showed two intramolecular interactions: one being a hydrogen bond between the aniline NH and the oxygen of the hydroxyamidine, and the other between the nitrogen of the hydroxyamidine and the NH of indazole. In our docking model, we identified that halogen bonding between Cys129 and the Tyr126 backbone improves the activity against IDO1. As a surface model of the active site, **8a** does not occupy the extended pocket B. We surmise that this could be an opportunity to further improve the potency for **8a** by structure-based design.

## 3. Materials and Methods

### 3.1. Chemistry

#### 3.1.1. General Chemistry

All materials were obtained from commercial suppliers and used without further purification. Solvents in this study were dried using an aluminum oxide column. Thin-layer chromatography was performed on pre-coated silica gel 60 F254 plates. Purification of intermediates was carried out by normal phase column chromatography (MPLC, Silica gel 230-400 mesh). NMR spectra were recorded on a Avance 300 MHz (Bruker, Billerica, MA, USA). LC/MS data were obtained using a Waters 2695 LC and Micromass ZQ spectrometer (Waters, Milford, MA, USA). HR-MS was detected on JMS-700 (JEOL, Tokyo, Japan). The identity of the final compounds was confirmed by proton NMR and mass spectrometry.

#### 3.1.2. General Procedure for the Preparation of Compounds

##### Synthesis of 1*H*-Indazol-7-amine **3**

A mixture of 7-nitroindazole (3 g, 0.018 mol)) and 10% Pd/C (0.6 g) in EtOH (10 mL) was stirred at rt under H_2_ atmosphere. The mixture was filtered through a pad of Celite and washed with EtOH. The filtrate was concentrated to give compound **3** (1.9 g, 78%, ivory solid).^1^H-NMR (300 MHz, CDCl_3_) δ 8.09 (s, 1H), 7.24 (d, *J* = 8.2 Hz, 1H), 7.10*–*6.96 (m, 1H), 6.68 (d, *J* = 7.2 Hz, 1H), 4.12 (brs, 1H).

##### Synthesis of 7-Iodo-1*H*-indazole **4**

To a solution of **3** (0.3 g, 2.25 mmol) in c-HCl (0.56 mL) and 98% H_2_SO_4_ (2 mL) was added dropwise a solution of NaNO_2_ (0.154 g, 2.23 mmol) in H_2_O (2 mL). The mixture was stirred at 0 °C for 30 min. Then, a solution of KI (0.747 g, 4.5 mmol) in H_2_O (2 mL) was added dropwise and the mixture was stirred at rt for 2 h. The mixture was diluted with EA, washed with sat. NaHCO_3_, water, and brine. The organic layer was dried over MgSO_4_ and purified by column chromatography (MPLC) to give compound **4** (0.17 g, 32%, dark ivory solid). ^1^H-NMR (300 MHz, CDCl_3_) δ 10.14 (s, 1H), 8.26 (s, 1H), 7.82*–*7.71 (m, 2H), 7.04*–*6.92 (m, 1H).

##### Synthesis of 1*H*-Indazole-7-carbonitrile **5**

A solution of **4** (0.3 g, 1.23 mmol), dppf (55 mg, 0.098 mmol), Pd_2_(dba)_3_ (28 mg, 0.049 mmol), Zn (20 mg, 0.307 mmol), Zn(CN)_2_ (86.6 mg, 0.737 mmol) in DMA (30 mL, degassed) was heated at 120 °C for 5 h. The mixture was concentrated and diluted with EA. The organic layer was washed with 2 N NH_4_OH and brine. The organic layer was dried over MgSO_4_ and concentrated to obtain the crude mixture which was purified by column chromatography (MPLC) to give compound **5** (173 mg, 98%, white solid). ^1^H-NMR (300 MHz, CDCl_3_) δ 11.85 (s, 1H), 8.41 (s, 1H), 8.09 (dd, *J* = 8.2, 0.9 Hz, 1H), 7.81 (dd, *J* = 7.3, 0.9 Hz, 1H), 7.36*–*7.30 (m, 1H).

##### Synthesis of (*Z*)-*N′-*Hydroxy-1*H*-indazole-7-carboximidamide **6**

To a solution of **5** (0.1 g, 0.7 mmol) in H_2_O (1.5 mL)/EtOH (3 mL) were added hydroxylamine hydrogen chloride (0.073 g, 1.05 mmol) and sodium bicarbonate (0.118 g, 1.4 mmol) and heated at 85 °C for 8 h. The reaction mixture was diluted with EA, washed with water and brine. The organic layer was dried over MgSO_4_ and concentrated to give compound **6** (84 mg, 68%, white solid) ^1^H-NMR (300 MHz, DMSO) δ 12.22 (s, 1H), 9.82 (s, 1H), 8.12 (s, 1H), 7.76 (dd, *J* = 20.6, 7.5 Hz, 2H), 7.17 (t, *J* = 7.5 Hz, 1H), 6.07 (s, 2H).

##### Synthesis of (*E*)-*N*-Hydroxy-1*H*-indazole-7-carbimidoyl chloride **7**

To a suspension of 6 (74 mg, 0.42 mmol) in water (1 mL) were added acetic acid (0.5 mL) and 6 N HCl (1.26 mmol, 0.21 mL) and this suspension was stirred at 45 °C until complete solution was achieved. Sodium chloride (0.074 g, 1.26 mmol) was added to the reaction mixture and stirred in an ice/water/methanol bath. A solution of NaNO_2_ (0.029 g, 0.42 mmol) in water (1 mL) was added dropwise while maintaining the temperature below 0 °C. After complete adding stirring was continued in the ice bath for 1.5 h and then the mixture was allowed to warm to 15 °C. The precipitate was collected by filtration and washed with cold water to give the desired compound **7** (65 mg, 79% ivory solid) ^1^H-NMR (300 MHz, DMSO-*d*_6_) δ 12.74 (s, 1H), 12.47 (s, 1H), 8.23 (s, 1H), 7.94 (d, *J* = 8.1 Hz, 1H), 7.82 (dd, *J* = 7.5, 0.6 Hz, 1H), 7.29 (t, *J* = 3.75 Hz, 1H).

##### General Procedure for the Synthesis of **8**, **12** and **16**

(*Z*)*-N-*(*3-Bromo-4-fluorophenyl*)*-N′-hydroxy-1H-indazole-7-carboximidamide* (**8a**)*.* To a solution of **7** (25 mg, 0.105 mmol) in THF (1 mL) at 60 °C was 3-bromo-4-fluoroaniline (20 µL, 0.105 mmol) was added and stirred for 10 min. A solution of NaHCO_3_ (13 mg, 0.157 mmol) in water (1 mL) was added dropwise and stirred at 60 °C for 3 h. The mixture was extracted with EA and washed with brine. The organic layer was dried over MgSO_4_ and concentrated to obtain the crude mixture which was purified by column chromatography (MPLC) to give compound **8a**. ^1^H-NMR (300 MHz, CDCl_3_) δ 12.01 (s, 1H), 8.14 (s, 1H), 7.73 (d, *J* = 8.0 Hz, 1H), 7.48 (s, 1H), 7.20 (d, *J* = 7.2 Hz, 1H), 7.01 (t, *J* = 7.7 Hz, 1H), 6.95 (dd, *J* = 5.7, 2.3 Hz, 1H), 6.78 (t, *J* = 8.4 Hz, 1H), 6.58*–*6.43 (m, 1H); LC/MS (ESI) *m*/*z* 349 [M + H]^+^; HRMS (EI) *m*/*z* calcd. for C14H10BrFN4O [M^+^] 348.0022, found 348.0019.

(*Z*)*-N-*(*3-Chloro-4-fluorophenyl*)*-N′-hydroxy-1H-indazole-7-carboximidamide* (**8b**)*.* ^1^H-NMR (300 MHz, CDCl_3_) δ 11.96 (brs, 1H), 8.15 (s, 1H), 7.74 (d, *J* = 8.0 Hz, 1H), 7.44 (s, 1H), 7.22 (d, *J* = 7.2 Hz, 1H), 7.06*–*6.94 (m, 1H), 6.85*–*6.75 (m, 2H), 6.53*–*6.39 (m, 1H); LC/MS (ESI) *m*/*z* 305 [M + H]^+^ ; HRMS (EI) *m*/*z* calcd. for C_14_H_10_C_l_FN_4_O [M^+^] 304.0527, found 304.0519.

(*Z*)*-N-*(*3-Chlorophenyl*)*-N′-hydroxy-1H-indazole-7-carboximidamide* (**8c**)*.* ^1^H-NMR (300 MHz, CDCl_3_) δ 11.91 (s, 1H), 8.16 (s, 1H), 7.74 (d, *J* = 8.0 Hz, 1H), 7.43 (s, 1H), 7.34*–*7.25 (m, 1H), 7.08*–*6.94 (m, 2H), 6.90 (d, *J* = 7.9 Hz, 1H), 6.77 (s, 1H), 6.51 (d, *J* = 7.8 Hz, 1H); LC/MS (ESI) *m*/*z* 287 [M + H]^+^; HRMS (EI) *m*/*z* calcd. for C_14_H_11_C_l_N_4_O [M^+^] 286.0621, found 286.0627.

(*Z*)*-N-*(*4-Fluorophenyl*)*-N′-hydroxy-1H-indazole-7-carboximidamide* (**8d**)*.* ^1^H-NMR (300 MHz, CDCl_3_) δ 11.98 (s, 1H), 8.15 (s, 1H), 7.71 (dd, *J* = 8.0, 0.7 Hz, 1H), 7.43 (s, 1H), 7.21 (dd, *J* = 7.3, 0.7 Hz, 1H), 7.02*–*6.95 (m, 1H), 6.82*–*6.74 (m, 2H), 6.72*–*6.63 (m, 2H); LC/MS (ESI) *m*/*z* 271 [M + H]^+^.

(*Z*)*-N-*(*2,5-Difluorophenyl*)*-N′-hydroxy-1H-indazole-7-carboximidamide* (**8e**)*.* ^1^H-NMR (300 MHz, CDCl_3_) δ 8.21 (s, 1H), 7.83 (dd, *J* = 8.1, 0.8 Hz, 1H), 7.46*–*7.39 (m, 1H), 7.18 (s, 1H), 7.17*–*7.10 (m, 1H), 7.10*–*6.99 (m, 1H), 6.66*–*6.53 (m, 1H), 6.36*–*6.24 (m, 1H); LC/MS (ESI) *m*/*z* 289 [M + H]^+^.

(*Z*)*-N-*(*3-Acetylphenyl*)*-N′-hydroxy-1H-indazole-7-carboximidamide* (**8f**)*.* ^1^H-NMR (300 MHz, DMSO) δ 12.80 (s, 1H), 10.79 (s, 1H), 8.64 (s, 1H), 8.09 (s, 1H), 7.78 (d, *J* = 7.9 Hz, 1H), 7.35–7.27 (m, 3H), 7.16*–*7.04 (m, 2H), 6.88 (d, *J* = 9.5 Hz, 1H), 2.31 (s, 3H); LC/MS (ESI) *m*/*z* 295 [M + H]^+^.

(*Z*)*-N′-Hydroxy-N-*(*m-tolyl*)*-1H-indazole-7-carboximidamide* (**8g**)*.* ^1^H-NMR (300 MHz, CDCl_3_) δ 11.80 (s, 1H), 8.17 (s, 1H), 7.74 (dd, *J* = 8.1, 0.8 Hz, 1H), 7.37*–*7.27 (m, 2H), 7.11*–*6.95 (m, 2H), 6.79 (d, *J* = 7.5 Hz, 1H), 6.66 (s, 1H), 6.54 (d, *J* = 7.9 Hz, 1H), 2.21 (s, 3H); LC/MS (ESI) *m*/*z* 267 [M + H]^+^.

(*Z*)*-N-*(*4-Chloro-3-methylphenyl*)*-N′-hydroxy-1H-indazole-7-carboximidamide* (**8h**)*.* ^1^H-NMR (300 MHz, DMSO) δ 12.77 (s, 1H), 10.75 (s, 1H), 8.44 (s, 1H), 8.10 (d, *J* = 1.3 Hz, 1H), 7.78 (d, *J* = 7.9 Hz, 1H), 7.26 (d, *J* = 6.3 Hz, 1H), 7.12*–*7.03 (m, 1H), 6.99 (d, *J* = 8.6 Hz, 1H), 6.74 (d, *J* = 2.6 Hz, 1H), 6.39 (dd, *J* = 8.5, 2.7 Hz, 1H), 2.10 (s, 3H); LC/MS (ESI) *m*/*z* 301 [M + H]^+^.

(*Z*)*-N-*(*3-Chloro-4-methoxyphenyl*)*-N′-hydroxy-1H-indazole-7-carboximidamide* (**8i**)*.* ^1^H-NMR (300 MHz, CDCl_3_) δ 12.0 (brs, 1H), 8.13 (s, 1H), 7.70 (d, *J* = 8.0 Hz, 1H), 7.40 (s, 1H), 7.20 (d, *J* = 7.3 Hz, 1H), 6.98 (t, *J* = 7.7 Hz, 1H), 6.86 (d, *J* = 2.6 Hz, 1H), 6.58 (d, *J* = 8.8 Hz, 1H), 6.49 (dd, *J* = 8.8, 2.6 Hz, 1H), 3.78 (s, 3H); LC/MS (ESI) *m*/*z* 317 [M + H]^+^.

(*Z*)*-N′-Hydroxy-N-phenyl-1H-indazole-7-carboximidamide* (**8j**)*.* ^1^H-NMR (300 MHz, CDCl_3_) δ 11.59 (brs, 1H), 8.18 (s, 1H), 7.76 (d, *J* = 8.0 Hz, 1H), 7.34 (d, *J* = 7.2 Hz, 2H), 7.16 (t, *J* = 7.6 Hz, 2H), 7.08*–*6.95 (m, 2H), 6.81 (d, *J* = 7.8 Hz, 2H); LC/MS (ESI) *m*/*z* 253 [M + H]^+^.

(*Z*)*-N-*(*4-Chlorophenyl*)*-N′-hydroxy-1H-indazole-7-carboximidamide* (**8k**)*.* ^1^H-NMR (300 MHz, CDCl_3_) δ 11.71 (brs, 1H), 8.18 (s, 1H), 7.77 (d, *J* = 8.0 Hz, 1H), 7.37*–*7.25 (m, 3H), 7.10 (d, *J* = 8.5 Hz, 2H), 7.06 (t, *J* = 7.7 Hz, 1H), 6.71 (d, *J* = 8.5 Hz, 2H); LC/MS (ESI) *m*/*z* 287 [M + H]^+^.

(*Z*)*-N′-Hydroxy-N-*(*4-methoxyphenyl*)*-1H-indazole-7-carboximidamide* (**8l**)*.* ^1^H-NMR (300 MHz, CDCl_3_) δ 11.95 (s, 1H), 8.14 (s, 1H), 7.70 (d, *J* = 7.9 Hz, 1H), 7.37 (s, 1H), 7.23 (d, *J* = 7.1 Hz, 1H), 6.97 (t, *J* = 7.7 Hz, 1H), 6.72 (d, *J* = 9.0 Hz, 2H), 6.65 (d, *J* = 8.9 Hz, 2H), 3.72 (s, 3H); LC/MS (ESI) *m*/*z* 283 [M + H]^+^.

(*Z*)*-N′-Hydroxy-N-*(*p-tolyl*)*-1H-indazole-7-carboximidamide* (**8m**)*.* ^1^H-NMR (300 MHz, CDCl_3_) δ 11.53 (s, 1H), 8.17 (s, 1H), 7.75 (d, *J* = 8.1 Hz, 1H), 7.32 (d, *J* = 6.6 Hz, 1H), 7.27 (s, 1H), 7.07*–*6.99 (m, 1H), 6.97 (d, *J* = 8.1 Hz, 2H), 6.73 (d, *J* = 8.3 Hz, 2H), 2.27 (s, 3H); LC/MS (ESI) *m*/*z* 267 [M + H]^+^.

##### Synthesis of *N-*Hydroxy-1-methyl-1H-indazole-7-carbimidoyl chloride (**11**)

A solution of 1-methyl-1H-indazole-7-carbaldehyde oxime **10** (0.1 g, 0.57 mmol), NCS (0.076 g, 0.57 mmol) in DMF was stirred at rt for 6 h. The mixture was diluted with EA and washed with water and brine. The organic layer was dried over MgSO_4_ and concentrated to give the crude product **11**. ^1^H-NMR (300 MHz, CDCl_3_) δ 10.11 (s, 1H), 8.08 (s, 1H), 7.84 (dd, *J* = 8.1, 0.9 Hz, 1H), 7.58 (dd, *J* = 7.2, 0.9 Hz, 1H), 7.24*–*7.19 (m, 1H), 4.15 (s, 3H).

(*Z*)*-N-*(*3-Bromo-4-fluorophenyl*)*-N′-hydroxy-1-methyl-1H-indazole-7-carboximidamide* (**12**)*.* ^1^H-NMR (300 MHz, CDCl_3_) δ 8.02 (s, 1H), 7.82 (dd, *J* = 8.1, 1.0 Hz, 1H), 7.60 (br, 1H), 7.35 (dd, *J* = 7.1, 1.0 Hz, 1H), 7.20*–*7.11 (m, 1H), 6.82*–*6.75 (m, 1H), 6.73*–*6.64 (m, 1H), 6.38*–*6.29 (m, 1H), 4.09 (d, *J* = 6.2 Hz, 3H); LC/MS (ESI) *m*/*z* 363 [M + H]^+^.

(*Z*)*-N-*(*3-Bromo-4-fluorophenyl*)*-N′,2-dihydroxybenzimidamide* (**16**)*.* ^1^H-NMR (300 MHz, CDCl_3_) δ 7.27*–*7.18 (m, 1H), 7.13*–*7.07 (m, 1H), 7.07*–*6.98 (m, 2H), 6.98*–*6.89 (m, 1H), 6.76*–*6.65 (m, 2H); LC/MS (ESI) *m*/*z* 325 [M + H]^+^.

### 3.2. Biology

#### 3.2.1. Generation of Human Ido1 Gene Expressing Hek293 Recombinant Cells

cDNA of human IDO1 gene (provided from Korean UniGene, Daejeon, Korea) was inserted into pcDNA5/FRT/TO expression vector (Invitrogen, Waltham, MA, USA) and transfected into Flp-In-Rex- HEK293 cells (Invitrogen). After transfection, homogenous IDO1 expressing HEK293 cells were selected using hygromycin and confirmed for their IDO1 expression using Western blot.

#### 3.2.2. Cell Based Assay for Analysis of Anti-Ido1 Activity of Compounds by Determination of Tryptophan and Kynurenine Using an LC-MS System

To analyze anti-IDO1 activity of compounds, human IDO1 expressing HEK293 recombinant cells were seeded in 100 μL of complete MEM media containing 10% FBS in a 96-well plate. After incubation for 48 hours, various concentrations of compounds with 0.2 ng/mL doxycyclin (Sigma Aldrich, St. Louis, MO, USA) was treated and incubated for 24 h. Culture supernatants were collected and prepared for analysis of tryptophan and kynurenine using LC-MS. For preparation of samples for LC-MS analysis, 30 μL of culture supernatant was mixed with 2 μM caffeine containing 270 μL methanol. After centrifugation at 12,000 rpm for 10 min, 100 μL of supernatant was transferred into new 1.5 mL tubes. Distilled water (900 μL) was added into 100 μL of supernatant and vortexed. The concentrations of tryptophan and kynurenine were determined by an LC-MS/MS system consisting of an Agilent 1200 series HPLC system (Agilent Technologies, Santa Clara, CA, USA) with an API 4000 QTRAP hybrid triple quadrupole/linear ion trap mass spectrometer (AB Sciex, Foster City, CA, USA) equipped with a turbo electrospray interface. Chromatographic separation of tryptophan and kynurenine was achieved on a Unison UK-C_18_ column (100 mm × 2 mm i.d., 3 μm; Imtakt, Kyoto, Japan) with a Security Guard C_18_ guard column (4 mm × 20 mm i.d.; Phenomenex, Torrance, CA, USA) maintained at 25 °C. Isocratic mode was used to achieve separation of the analytes using mobile phase (acetonitrile with 0.1 formic acid and water with 0.1% formic acid (20:80 *v*/*v*) at a flow rate of 300 μL/min for 3 min. The multiple reaction monitoring (MRM) transition was *m*/*z* 204.94 → 188.1 for tryptophan and *m*/*z* 208.97 → 192.00 for kynurenine under positive-ionization. Optimized values for de-clustering potential, collision energy, entrance potential, and collision-exit potential were 26 V, 15 eV, 9.5 V and 8 V and 21 V, 13 eV, 10 V and 8 V for tryptophan and kynurenine, respectively. The optimized instrument conditions were as follows: ion spray voltage, 5500 V; source temperature, 450 °C; curtain gas, 25 psi; nebulizing gas (GS1), 30 psi; heating gas (GS2), 30 psi. Data analysis was performed using Analyst Software version 1.4.2. (AB Sciex, Foster City, CA, USA).

#### 3.2.3. hIDO1 Enzymatic Assay

To analyze the hIDO1 inhibitory activities of compounds, we used IDO1 inhibitor screening assay kit and followed the protocol provided from BPS Bioscience (San Diego, CA, USA). Briefly, 5 μL of IDO solution was aliquoted into a 384-well plate and treated with 5 μL of compound in buffer at room temperature for 3 h. The data was measured at *λ* = 320 to 325 nm.

### 3.3. Docking Study

Molecular modeling study of **8a** against IDO1 was performed using the Schrödinger Suite 2017-2 (Schrödinger, LLC, New York, NY, USA) [22]. The X-ray crystal structure of IDO1 was obtained from the Protein Data Bank [23]. The IDO1 protein preparation was revised using Protein Preparation Wizard in Maestro v.11.2. The receptor grid box was generated 25 × 25 × 25 Å cubic size centered on complexed heme. The compound was minimized using an OPLS_2005 force field with a dielectric constant value 80.0 in MacroModel v.11.6. The flexible docking of **8a** was performed using the Standard Precision method in Glide v.7.5 with positional constraints for covalent bonding with heme iron. The docking model of **8a** was visualized using Discovery Studio 2016 (Biovia, San Diego, CA, USA) [24].

## 4. Conclusions

In conclusion, we designed and synthesized a series of *N′*-hydroxyindazolecarboximidamides for inhibition of IDO1. Among the compounds synthesized, compound **8a** exhibited moderate IDO1 inhibitory activity in both tryptophan depletion and kynurenine production assays. In addition, the molecular docking study with compound **8a** suggested a structural basis for its IDO1 inhibition. Although IDO1 inhibitory activities are relatively lower than those of known inhibitors, an indazole scaffold might be a useful novel structure for further optimization study as an IDO1 inhibitor.

## Figures and Tables

**Table 1:**
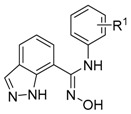
Inhibition of tryptophan depletion and kynurenine production through IDO1.

No.	R^1^	HEK293 IC_50_ (µM)		hIDO1 Inhibition at 20 µM (%)
		Tryptophan Depletion	Kynurenine Production	
**8a**	3-Br-4-F	1.76	2.13	81
**8b**	3-Cl-4-F	3.26	3.14	79
**8c**	3-Cl	4.95	4.89	77
**8d**	4-F	>10	>10	n.t.
**8e**	2,5-diF	>10	>10	n.t.
**8f**	3-Ac	>10	>10	n.t.
**8g**	3-Me	>10	>10	n.t.
**8h**	4-Cl-3-Me	>10	>10	n.t.
**8i**	3-Cl-4-MeO	>10	>10	n.t.
**8j**	H	>10	>10	n.t.
**8k**	4-Cl	>10	>10	n.t.
**8l**	4-MeO	>10	>10	n.t.
**8m**	4-Me	>10	>10	n.t.
**12**	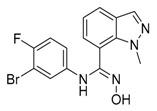	>10	>10	15
**16**	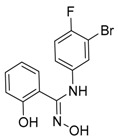	7.76	7.91	n.t.

n.t. = not tested.
